# *Drawing as a Way of Knowing*: How a Mapping Model Assists Preoperative Evaluation of Patients with Thyroid Carcinoma †

**DOI:** 10.3390/jcm13051389

**Published:** 2024-02-28

**Authors:** Marco Biffoni, Giorgio Grani, Rossella Melcarne, Valerio Geronzi, Fabrizio Consorti, Giuseppe De Ruggieri, Alessia Galvano, Maryam Hosseinpour Razlighi, Eva Iannuzzi, Tal Deborah Engel, Daniela Pace, Cira Rosaria Tiziana Di Gioia, Marco Boniardi, Cosimo Durante, Laura Giacomelli

**Affiliations:** 1 Department of General and Specialist Surgery, Sapienza University of Rome, Viale del Policlinico, 155, 00161 Rome, Italy; marco.biffoni@uniroma1.it (M.B.); geronzi.1785528@studenti.uniroma1.it (V.G.); giuseppe.deruggieri@libero.it (G.D.R.); alessia.galvano@uniroma1.it (A.G.); maryam.mhpour@yahoo.com (M.H.R.); evaiannuzzi1998@gmail.com (E.I.); tal29engel@gmail.com (T.D.E.); laura.giacomelli@uniroma1.it (L.G.); 2 Department of Translational and Precision Medicine, Sapienza University of Rome, Viale del Policlinico, 155, 00161 Rome, Italy; giorgio.grani@uniroma1.it (G.G.); cosimo.durante@uniroma1.it (C.D.); 3 Department of General Surgery, Sapienza University of Rome, Viale del Policlinico, 155, 00161 Rome, Italy; fabrizio.consorti@uniroma1.it; 4 Department of Endocrinology, Valmontone Hospital, 00038 Valmontone, Italy; dani.pace@tiscalinet.it; 5 Department of Radiological, Oncological and Pathological Sciences, Sapienza University of Rome, Viale Regina Elena 324, 00161 Rome, Italy; cira.digioia@uniroma1.it; 6 Endocrine Surgery Unit, Niguarda Hospital, 20162 Milan, Italy; marco.boniardi@ospedaleniguarda.it

**Keywords:** ultrasonography, performance, prediction, central compartment, lymph node, radical neck dissection, extent of resection, multidisciplinary approach, thyroid unit

## Abstract

**Background**: Effective pre-surgical planning is crucial for achieving successful outcomes in endocrine surgery: it is essential to provide patients with a personalized plan to minimize operative and postoperative risks. **Methods:** Preoperative lymph node (LN) mapping is a structured high-resolution ultrasonography examination performed in the presence of two endocrinologists and the operating surgeon before intervention to produce a reliable “anatomical guide”. Our aim was to propose a preoperative complete model that is non-invasive, avoids overdiagnosis of thyroid microcarcinomas, and reduces medical expenses. **Results:** The use of ‘preoperative echography mapping’ has been shown to be successful, particularly in patients with suspected or confirmed neoplastic malignancy. Regarding prognosis, positive outcomes have been observed both post-surgery and in terms of recurrence rates. We collected data on parameters such as biological sex, age, BMI, and results from cytologic tests performed with needle aspiration, and examined whether these parameters predict tumor malignancy or aggressiveness, calculated using a multivariate analysis (MVA). **Conclusions:** A standard multidisciplinary approach for evaluating neck lymph nodes pre-operation has proven to be an improved diagnostic and preoperative tool.

## 1. Introduction

It has been demonstrated that high-resolution ultrasonographic examination can detect thyroid nodules in 19–68% of individuals [1], as 7–15% of those nodules turn out to be malignant. Differentiated thyroid carcinoma (DTC) accounts for 90% of thyroid neoplasms, most of which are papillary thyroid carcinomas (PTC) [2].

Loco-regional lymph node metastasis (LNM) is highly common in differentiated thyroid carcinomas, estimated at approximately 30 to 80% of cases, depending on the method used for detecting them [3].

The presence of LNM is the most common risk factor for disease recurrence. In most cases, disease recurrence appears in the first 5 years after thyroidectomy surgery, meaning an undetected lymph node metastasis was not treated [3].

High-resolution ultrasound (HDUS) examination is the first-line diagnostic imaging method for preoperative evaluation of LMN in PTC. With HDUS, LNM as small as 5 mm can be detected [4].

To date, one of the major challenges in both clinical and surgical thyroid nodule management is to avoid overtreatment in patients with low disease activity and to be able to diagnose clinically advanced tumors that progress rapidly. In fact, thyroid carcinomas are often present as an established cancerous mass or subclinical LNM when diagnosed (up to 80% of patients with PTC) [5].

The American Association of Endocrine Surgeons (AAES) guidelines for surgical management of adult thyroid disease [2], published in 2020, strongly recommend routine preoperative bilateral ultrasound (US) evaluation of LN compartments II-VI of patients with cytologically evident thyroid carcinoma. Such evaluation could be extended to levels I-VII in patients where metastasis has been identified (the “mapping” technique) [2,6].

Following the AAES guidelines, our department developed the mapping technique into a model that allows surgeons to prepare for surgery in a noninvasive, visually adjusted, rapid, and less expensive manner, which improved post-operative results, especially regarding recurrence.

### Aim of the Study

In this study, we intended to show how our technique of mapping, combining HDUS (performed in accordance with the AAEG guideline) and the endocrinological written report into a graphic representation, had formed a complete and comprehensive pre-operation model. We were able, furthermore, to obtain better results in patients’ post-operation. Surgery became more accurate, and unnecessary nodular dissection (ND) was significantly reduced.

We also aimed to investigate whether there is an alternative to the written report and the HDUS that could give better predictions while evaluating patients assigned to undergo an operation. We wanted to check if there was a correlation between certain characteristics of patients that could influence the preoperative assessment of nodal metastasis.

Throughout the study, we examined:

1. Both the adequacy and accuracy of US exploration of the LN, as they were observed throughout the process of mapping.
2. Personal characteristics of the patient that could predict LN malignancy or provide additional information to the US, such as age, sex, and BMI, especially regarding the malignancy of the nodules.

## 2. Materials and Methods

### 2.1. Inclusion Criteria

We carried out a retrospective real-life observational study at the Dept. of General and Specialist Surgery and the Dept. of Translational and Precision Medicine of Sapienza University of Rome. Patients met the following inclusion criteria: (a) thyroid surgery for sonographic and/or cytologically suspicious thyroid nodules; (b) complete histopathological report; (c) one pre-surgical neck ultrasound with available images and report, carried out as a *preoperative mapping*; and (d) neck US 6–12 months after surgery (for locoregional surveillance; check for persistence and recurrence disease [6,7,8]).

These inclusion criteria are based on a routine agreed-upon protocol.

### 2.2. Preoperative Mapping Procedure

**Figure 1:**
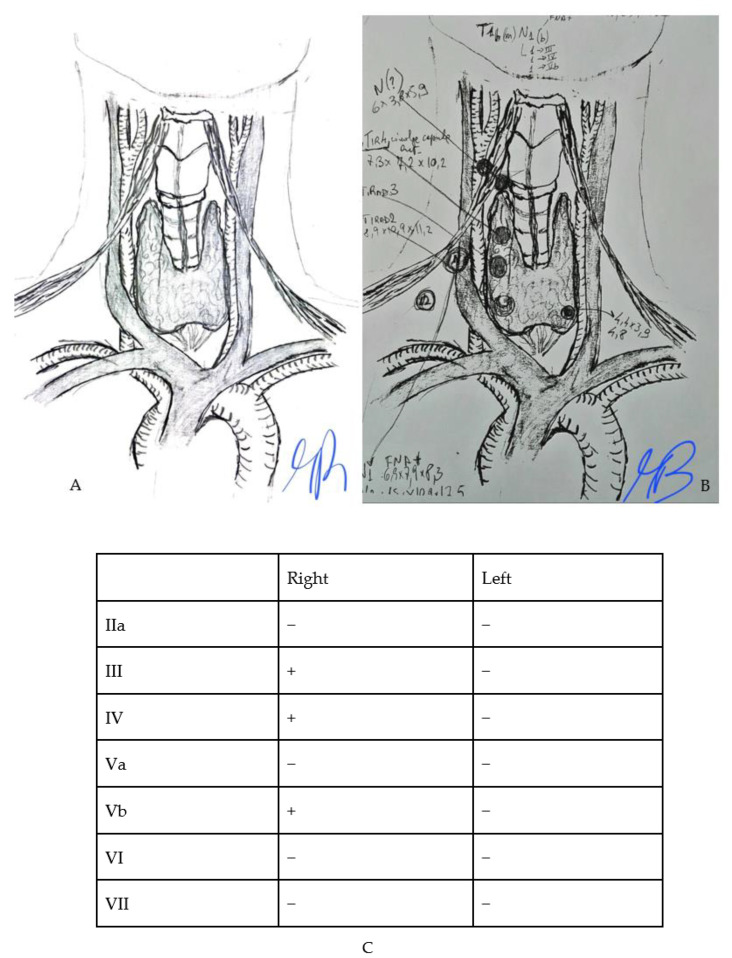
Example of model-tool used for *preoperative mapping* (**A**) the original template (**B**) an example of *preoperative* drawing on the model (**C**) an example of a table that is drawn to indicate the presence of lymph nodes neoplasms.

**Figure 2:**
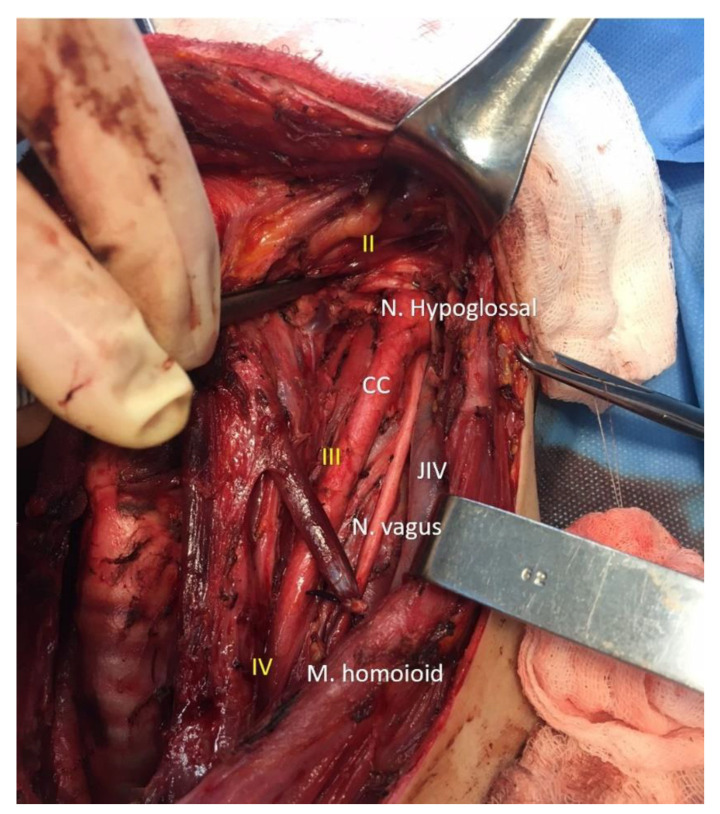
Intraoperative image with lymph node levels and key vascular-nervous structures of the neck. CC: common carotid artery. JIV: internal jugular vein. Nerve hypoglossal, Nerve vagus, Muscle homoioid. II lymph node level in the neck. III lymph node level in the neck. IV lymph node level in the neck.

All recruited subjects had undergone, the day before surgery, a high-resolution US examination of the neck (*preoperative mapping*) performed by an endocrinologist for the evaluation of US features of suspected thyroid nodules and targeted the evaluation of neck LN and their characteristics. A second endocrinologist, an ultrasound specialist, and the surgeon attended the procedure. The two endocrinologists reached consensus decisions to reduce inter-observer variability. During the examination, a resident surgeon drew an anatomical model of the neck on paper, marking the exact location of the suspected LN reported while mapping (Figure 1), and the reported size. Finally, the presence or absence of suspected LN was put in a summary table (Figure 1C). This graphic illustration provided the surgeon with the exact location of the suspected LN, making surgery as accurate as possible. At the end of the procedure, the clinical case was discussed among healthcare providers and the surgical plan was then communicated to the patient. Surgical procedures were performed between November 2015 and June 2023 by two senior endocrine surgeons (>100 thyroidectomies/year) (Figure 2).

### 2.3. Thyroid Nodule Evaluation

Sonographic examinations were performed following a uniform protocol. A high-frequency linear probe was used, adjusting the frequency according to the depth of the field explored and with the integration of color flow or power Doppler examination when necessary, presenting nodal vascularization. During the US mapping, the examiner paid special attention to detecting any extrathyroidal extension, multifocality, and features of the nodule undergoing needle aspiration.

The following data were collected for US assessment:

- Size, expressed in mm.
- Composition.
- Echogenicity.
- Margin.
- Shape/direction of growth.
- Echogenic foci/calcification.
- Extrathyroidal extension.
- Lymph nodes.

We used an international consensus lexicon for the US description of nodules and a standardized report, according to the European Thyroid Association Guidelines for ultrasound malignancy risk stratification of thyroid nodules in adults, the EU-TIRADS [9].

### 2.4. Neck Lymph Nodes Evaluation

Ultrasonographic features labeling lymph nodes as suspicious were standardized and recorded to reduce differences among operators [8].

The patient lay on a couch with the neck well extended, in a position that makes exploration of the central VI and IV levels of the neck easier and more accessible. Three regions were scanned separately: the central level (VI), levels IIA to IV inclusive, and levels VA and VB.

Lateral and central cervical compartments were evaluated in the transverse plane, while identified abnormalities were imaged in the longitudinal plane and evaluated by Doppler.

The probe was angled toward the sternal notch to evaluate the upper mediastinum. Since ultrasound cannot adequately present the deeper compartments of the neck, such as the retro- or parapharyngeal regions, evaluation of the tracheo-esophageal groove can be facilitated if the patient’s head is turned to the side.

For all LN compartments, any indeterminate or suspected LN was described, particularly their anatomic location (level), using the terminology and classification used for ND [10].

To identify the anatomic location of LNs, the patient’s neck was in a neutral position. In addition, the three-dimensional structure, internal content, echogenicity, presence of calcifications, and Doppler US features were reported.

The LNs were then classified as (1) normal, (2) indeterminate, or (3) suspected of malignancy. Malignancy was suspected when at least one of the following features was present:

- Microcalcifications.
- Partially cystic appearance.
- Peripheral or diffusely increased vascularization.
- Hyperechoic tissue looking like the thyroid [8].

### 2.5. Data Analysis

US mapping performance was assessed using the final pathology report as a standard reference, using Fisher’s exact test to determine the statistical significance of US findings and estimating sensitivity, specificity, positive and negative predictive value (PPV and NPV), likelihood ratio (LR), and accuracy, each with 95% CI. Statistical analysis was performed with JASP software version 0.18.1 (2023) considering LC-LN and CC-LN separately. The sensitivity and specificity of mapping in detecting suspicious LNs were evaluated by dividing patients according to (a) weight: three weight classes were considered (Normal Weight, Overweight, and Class I Obesity), (b) sex (Male and Female), and (c) age: three age classes were considered (20–35, 36–60, and >60 years). These are all risk factors associated with a poorer prognosis, and so we tested the hypothesis that preoperative US mapping could have a different significance in these risk groups. A multivariate analysis was performed for the CC-LN and LC-LN considering factors known in the pre-surgical analysis: (a) BMI, (b) cytology of the main nodule (TIR Classification), (c) sonographic size of the nodule examined via needle aspiration (expressed in mm), and (d) sonographic characteristics of the nodule: echogenicity, component, and the presence or absence of hyperechogenic spots.

The study was conducted according to the guidelines of the Declaration of Helsinki and approved by the Ethics Committee of Sapienza University of Rome (protocol code 4233, 12 December 2016). Informed consent was received from all subjects involved in the study.

## 3. Results

### 3.1. Clinical and Pre-Surgical Features

The final cohort consisted of 105 patients with suspicious nodules candidate for surgical resection, aged 19 to 84 (median 50; IQR 37–61), mostly female (75%).

The data collected regarding the BMI of the enrolled patients are shown in Table 1, with a minimum value of 19.13, a maximum value of 44.53, and an average of 25.84.

Ninety-seven patients (approximately 92% of the sample) had a cytological examination with suspicious/strongly suspicious or deponent for thyroid neoplastic pathology: Tir 3 36.2%, Tir 4 27.6%, and Tir 5 28.6%; one patient showed an FNAC that was Tir1 (Table 1).

Patients with a negative thyroid cytology report suggesting a malignant neoplasm (No. 7), presented, as a surgical indication, “lymph node metastatic recurrence” (No. 6) or a strong suspicion of lymph node metastasis from thyroid carcinoma on ultrasound (n.1). (Table 1).

### 3.2. Results of Preoperative Mapping for Nodular Assessment

All patients underwent an ultrasound examination of the neck immediately before surgery (referred to as ‘mapping’) to assess:

- The size of the nodule on which the needle aspiration had been performed.
- The Sonographic features of the needle aspiration nodule.
- Whether a lymph node is suspicious for neoplastic metastasis.
- The size of the major lymph node observed for each lymph node level.

The results of nodule characteristics are shown in Table 1 and those regarding lymph nodes in Table 2.

Seventeen patients (16.2%) underwent hemithyroidectomy surgery, 45 patients (42.9%) total thyroidectomy, 37 (35.2%) thyroidectomy and lymph node dissection, and 6 patients (5.7%) underwent lymph-node dissection (Table 3).

Histological examination of the removed glands and lymph nodes reported 88 (83,8%) diagnoses of malignant lesions (40 multifocal carcinomas). In two of these patients, the final pathology did not confirm the diagnosis of malignancy of the main nodule, but incidental nodules were found, and 17 (16,2%) had benign lesions. The distribution of malignant diagnoses is summarized in Table 4.

### 3.3. Results from Performing a Preoperative Mapping and Multivariate Analysis

The evaluation of the diagnostic accuracy of mapping to predict lymph node metastasis was compared with the data available post-operation (neck ultrasound) and those obtained by histological examination. The results are summarized in Table 5.

Statistical evaluation of sensitivity and specificity showed differences only in sensitivity, in overweight patients, and in central compartment lymph nodes alone.

According to the results achieved by the multivariate analysis, for both compartments and for each parameter taken into consideration (BMI, cytological, major nodule size with needle aspiration, echogenicity, hyperechogenic spots, and nodule component), the *p*-value resulted in always being well above 0.05, meaning that none of the parameters considered individually can be an effective statistical predictor for the presence of metastatic lymph nodes (Table 6).

Finally, two models that included all the previous factors were generated but, again, neither yielded better results than those obtained by ultrasound mapping:

- Central compartment model: sensitivity (56.0%), specificity (87.3%), and PPV (66.7%) compared with the values obtained from ultrasound mapping alone (sensitivity 45.7%, specificity 95.6%, PPV 84.2%, NPV 77.4%).
- Model of the lateral cervical compartment: sensitivity (33.3%), specificity (95.4%), and PPV (62.5%) compared with values obtained from ultrasound mapping alone (sensitivity 100%, specificity 88.8%, PPV 71.9%, NPV 100%).

## 4. Discussion

### 4.1. Our Findings and the Literature

The results of our study were in accordance with the AAES guideline: preoperative US mapping of LN does more accurately locate suspicious LNs and assesses whether the neoplastic features are aggressive [2,11].

When the number of nodules detected is low and their proportions are substantial (palpable), having an endocrinologist’s detailed report describing the location and size of the nodules is crucial. Nevertheless, as we saw in patients that were operated on in our department, when there are numerous nodules and their size is almost undetectable, creating a graphic model based on both the information in the report and the results from the H-US enabled us to obtain better results even in complicated cases.

The attempt to achieve better pre-operation results has been the department’s goal for years [12,13]. We learned that the complete thyroid mapping that we have been using allows for less mediated communication with the operating surgeon regarding their preparation, a more distinctive analysis of the surgery, and an even better understanding of the patient regarding their medical situation.

### 4.2. The Challenge in Diagnosing Carcinoma in Different Compartments of the Thyroid

In 2020, in a study by Abbound B et al. [14] investigating the diagnostic accuracy of neck US in the detection of LNM from PTC, a sensitivity value of 85% for LC and 69% for CC and a specificity value of 65% for LC and 71% for CC was found. In our patient cohort, we reported a higher SP (95.6% vs. 71% for CC and 88.8% vs. 65% LC) and SE (100% vs. 85% for LC).

In our study, we highlighted that there is a high specificity using an ultrasound method in detecting LC-LN and CC-LN. Comparing preoperative US mapping for suspicion of LNM and the results of cytology tests showed that the former has a high specificity (SP) (95.6% for the CC and 88.8% for the LC) compared with a sensitivity (SE) of 45.7% for the CC and 100% for the LC (PPV: 84,2% for the CC, 71.9% for the LC, NPV: 77.4% for the CC, 100% for the LC). Samples were taken from patients treated in our department, and the results obtained from the analysis are consistent with those reported in the literature.

The low sensitivity value in our study seems to be related to the difficulty in exploring CC- LN via ultrasound. This is why combining diagnostic methods, such as those of US and CT, had been suggested to increase sensitivity for CC and LC. With that, CT requires the use of intravenous iodinated contrast for increased accuracy [15], making it a diagnostic tool reserved only for patients with suspected extensive lateral neck disease or for regions not accessible by US [2,15].

Another approach taken to improve the detection of metastasis in CC-LN could be the combination of US and elastosonography [16,17]. In recent years, radiologists have focused on the development and validation of radiomic ultrasound nomograms as preoperative assessments of LN status in PTC.

In a study from 2020, a nomogram based on Shear-Wave radiomics Elastography (SWE) offered improved preoperative staging in cervical LNs in PTC [17]. Jiang L. et al. developed a promising clinical-radiomic nomogram, providing an optimal preoperative prediction of LN metastatic disease in PTC by applying contrast-enhanced ultrasound (CEUS)-based radiomic analysis to predict LNM in PTC [18]. The sensitivity found in our sample during CC exploration, however, matched that reported in the study by Abbound et al. (71.4% vs. 69%) [14] and in a meta-analysis published in 2019, which included 19 studies of 4014 patients (sensitivity for CC 0.33). Zhao et al. concluded that preoperative ultrasound demonstrated low preoperative diagnostic reliability for detecting LNM in the central compartment (reduced sensitivity) while having good diagnostic efficiency for detecting LNM in the lateral compartment [19].

Regarding prophylactic CND, the AAES guidelines point out that in the absence of clinically US-evident LNM, there are no conclusive data showing that aggressive prophylactic extirpation of LN with microscopic metastatic disease improves PTC outcomes (since microscopic metastasis is usually resected in prophylactic CND [2,20,21]).

Analyzing the lateral compartment before operating is fundamental, especially due to its anatomical structures and its rather confined locations, while the central compartment is more evident and accessible in its totality even during surgery. This could mean, for example, that in the case of intraoperatively finding a suspicious LN, microscopic neck metastasis of the central compartment does not seem to have a significant impact on surgery outcome [22]. Nevertheless, evident LN disease in the lateral neck compartments of DTC is known to have a significant oncological impact, and operating on it increases patients’ survival chances.

As found in the literature, to address the problem of “mapping” lymph nodes that are suspicious in imaging but are clinically negative, it has been suggested to map the central [23] and lateral [24] LNs of the neck with carbon nanoparticles might help surgeons to detect suspicious LNs during surgery. Yet the method we propose is simple to perform, noninvasive, does not require the application of chemical tracers, is easily applicable as it requires only the expertise of a skilled endocrinologist sonographer collaborating with a surgeon, and—compared with more advanced radiological methods—is less expensive.

Multidisciplinary teams, especially in the field of oncology, have been proven to give better results in many aspects of patient management [25,26,27]. Collaboration of an expert team shows a positive outcome not only in thyroid carcinomas but also in other neoplastic diseases like breast cancer, predicting a greater chance of recovery by 18% in patients treated with such an approach [28].

### 4.3. Patients’ Characteristics and Whether They Affect the Preoperative Evaluation of Nodal Metastasis

We wanted to evaluate whether characteristics like age, sex, and BMI could be risk factors that predict tumor malignancy and aggressiveness. Higher BMI was found to be associated with increased tumor size and LN metastases; hence, there was a significant correlation with poor prognosis [29,30]. The subjects in our study had proven that such characteristics are, in fact, incapable of predicting LN malignancy. Therefore, we do not consider them to be valid information in pre-operation assessment (Table 5 and Table 6). We performed a multivariate analysis with variables such as BMI, results of cytological tests performed on the largest nodule via needle aspiration, the size of the nodule, their echogenicity, aspect, and the presence or absence of hyperechogenic spots. Thyroid “mapping” and its results were not considered in the analysis.

First, each *p*-value of every factor was calculated separately. The results for both the central and lateral cervical compartments showed that none of these factors could predict LN malignancy. During the second stage of the analysis, all previous statistical predictors were considered together, but as presented by the given results, the sensitivity and specificity values were not higher than those of the US mapping (LC: *mapping* Se 100%, Sp88.8%, PPV 71.9%, NPV 100%; *model*: Se 33.3%, Sp 95.4%, PPV 62.5%; CC LN: *mapping* Se 45.7%, Sp 95.6%, PPV 84.2%, NPV 77.4%; *model*: Se 56%, Sp 87.3%, PPV 66.7%).

The only value that exceeds that of the mapping is the sensitivity represented while only the central compartment was examined (56% vs. 45.7%). Yet this does not allow these indicators to be considered reliable since sensitivity in the central compartment remained low and was associated with an even lower sensitivity value in the study of the lateral cervical compartment (33%); hence, these predictors do not allow for the precise localization of LNs of the neck and are of no use to surgical planning.

### 4.4. Strengths of the Study

This retrospective study included patients treated for thyroid neoplastic pathology over a long period of time (2015–2023) and was conducted in a central facility with a high number of patients diagnosed with thyroid neoplasm. In addition, the sample examined encompassed a wide range of patients, which allowed us to obtain significant statistical results. To reduce interobserver variability, a factor known to compromise the sensitivity of the US examination, there was an accordance between the two operators. This simple model was shown to be more valuable in selecting candidates for lateral and/or central cervical lymphadenectomy, capable of restricting such operations and performing them only if necessary, reducing operating room occupancy, hospitalization, and possible pre- and postoperative complications, and lowering associated healthcare costs.

### 4.5. Limitations of the Study

Regarding the interpretation of the results obtained, first, the retrospective nature of the study might have compromised the collection of the entire range of diagnostic information of patients, mostly US examination. In some patients, for example, missing documentation could not be found. Secondly, the US examination itself has operator-dependent variability. Finally, increased healthcare costs were due to the required presence of two experienced sonographers.

## 5. Conclusions

Ultrasonographic examination (*preoperative mapping*) proves to be an effective instrument for the detection of lateral cervical lymph nodes suspected to be metastatic or to create a recurrence of thyroid carcinoma.

Using the ultrasound findings and drawing them graphically on an anatomical template gave surgeons the exact display of the involved compartments with targeted NDs and permitted a rapid, efficient, and accurate planning of the surgical procedure. As a result, we were able to reduce rates of locoregional recurrence and reinterventions. This model also allowed us to inform patients about the operation they were about to undergo in a simple manner, and therefore improved doctor–patient communication as well.

## Figures and Tables

**Table 1:** Clinical and sonographic features of the study cohort.

		N.	%
** Clinical Features **			
Gender, male		26	24.80%
Gender, female		79	75.20%
BMI (Classes)			
Normal weight		52	49.50%
Overweight		38	36.20%
Obesity I		11	10.50%
Obesity II		3	2.90%
Obesity III		1	1.00%
** Pre-Surgical Citology **			
Tir 1		1	1.00%
Tir 2		0	0.00%
Tir 3A/B		38	36.20%
Tir 4		29	27.60%
Tir 5		30	28.60%
Absent		7	6.70%
** Sonographic Features of Main Nodule **			
Extrathyroidal extension (ETE)		35	33.30%
Microcalcifications		31	29.50%
Vascularity	Pattern 1	6	5.70%
	Pattern 2	11	10.50%
	Pattern 3	51	48.60%
	N/A	37	35.20%
Margins	Defined	18	17.10%
	Lobulated	23	21.90%
	Irregular	11	10.50%
	Ragged	7	6.70%
	Blurred	8	7.60%
	N/A	38	36.20%
Halo	Hypoechogenic	19	18.10%
	Hyperechogenic	2	1.90%
	Not specified	84	80.00%
Hyperechogenic spots		42	40.00%
Composition	Solid	50	47.60%
	Mixed	10	9.50%
	Almost completely solid	23	21.90%
	Not specified	22	21.00%
Echogenicity	Hypoechogenic	57	54.30%
	Hyperechogenic	2	1.90%
	Isoecogen	39	37.10%
	N/A	7	6.70%

Unit of measure BMI: kg/m^2^; Normal weight: 18.5–24.9; Overweight: 25–29.9; Obesity class I: 30–34.9; Obesity class II: 35–39.9; Obesity class III: >40; Consensus Statement AIT, AME, SIE & SIAPEC-IAP for the Classification and Reporting of Thyroid Cytol (2014); Tir 1: non-diagnostic; Tir 3: risk-indeterminate; Tir 4: suspicious of malignancy; Tir 5: malignancy; N/A: Not specified/absent.

**Table 2:** Distribution of maximum lymph node sizes of the lateral cervical and central compartments and distribution of suspected lymph nodes observed with preoperative mapping.

**Maximum lymph-nodes size (mm) on US**		
	Right	Left
Level 1	19	26
Level 2	48.5	47
Level 3	37.3	33
Level 4	44.5	35.4
Level 5	17.6	42.6
Level 6	35.2	36.7
Level 7	28	13
** Suspected lymph-nodes (n.) on US **		
	Yes	No
Lateral-cervical Compartment (LC-LN)	34	71
Central Compartment (CC-LN)	20	85
Level 1	1	104
Level 2	10	95
Level 3	14	91
Level 4	25	80
Level 5	6	99
Level 6	20	85
Level 7	2	103

US: ultrasonography.

**Table 3:** Distribution of types of endocrine surgical procedure in the examined sample.

Endocrine Surgical Procedures Performed		
	N.	%
Hemithyroidectomy (HT)	17	16.20%
Total Thyroidectomy (TT)	45	42.90%
TT + Nodal dissection (ND)	37	35.20%
ND	6	5.70%

**Table 4:** The distribution of malignant diagnoses and extracapsular, muscular, and vascular invasiveness features observed at final histological examination.

Malignant Histopathology Diagnosis							
	Number	Multifocality	No Citology	TIR1	TIR3A/B	TIR4	TIR5
PTC	75	37	6	0	15	26	28
PTC (incidental nodule)	2	0	0	0	2	0	0
FTC	4	2	0	0	4	0	0
MTC	3	1	1	0	0	0	2
NIFTP *	4	0	0	0	4	0	0
** Histo-pathology features **							
						**N.**	**%**
ETE						46	43.80%
Muscle Infiltration						6	5.70%
Angioinvasion						13	12.40%

PTC: Papillary thyroid cancer; FTC: Follicular thyroid cancer; MTC: Medullary thyroid cancer. * NIFTP: Noninvasive follicular thyroid neoplasm with papillary-like nuclear features. Considered a low-risk neoplasm according to WHO 2022 classification. ETE: Extrathyroidal Extension.

**Table 5:** Performance of *preoperative mapping* for lateral cervical compartment and central compartment.

DIAGNOSTIC PERFORMANCE *PREOPERATIVE MAPPING*													
		Se	IC95%		Sp	IC95%		PPV	IC95%		NPV	IC95%	
**LC-LN**	All Patients	100%	85.20%	100%	88.80%	79.70%	94.70%	71.90%	53.30%	86.25%	100%	94.90%	100%
	Normal weight	100%	69.20%	100%	87.50%	73.20%	95.80%	67%	38.90%	88.20%	100%	90%	100%
	Obesity I	100%	15.80%	100%	88.90%	51.80%	99.70%	67%	9.43%	99.16%	100%	63.10%	100%
	Overweight	100%	71.50%	100%	88.90%	70.80%	97.60%	79%	49.20%	95.34%	100%	85.75%	100%
	Males	100%	75.30%	100%	66.70%	34.90%	90.10%	76.47%	50.10%	93.19%	100%	63.06%	100%
	Females	100%	69.20%	100%	92.60%	83.70%	97.60%	66.70%	38.90%	88.20%	100%	94.31%	100%
	Age 20–35	100%	59.00%	100%	86.70%	59.50%	98.30%	77.80%	40%	97.20%	100%	75.30%	100%
	Age 36–60	100%	76.80%	100%	87.80%	73.80%	95.90%	73.70%	49%	90.10%	100%	90.30%	100%
	Age > 60	100%	15.80%	100%	91.30%	72.00%	98.90%	50%	6.80%	93.24%	100%	83.90%	100%
**CC-LN**	All Patients	45.70%	28.80%	63.40%	95.60%	87.60%	99.10%	84.20%	60.42%	96.62%	77.40%	67%	86%
	Normal weight	33.30%	13.30%	59.00%	96.90%	83.80%	99.90%	85.71%	42.12%	99.64%	72.10%	56.33%	84.70%
	Obesity I class	0.00%	0.00%	84.20%	88.90%	51.80%	99.70%	0.00%	0.00%	97.50%	80.00%	44.40%	97.50%
	Overweight	71.40%	41.90%	91.60%	95.80%	78.90%	99.90%	91%	59%	99.77%	85%	66.30%	95.80%
	Males	76.90%	46.20%	95.00%	75.00%	42.80%	94.50%	76.92%	46.20%	94.96%	75%	42.81%	94.51%
	Females	27.30%	10.70%	50.20%	100%	93.60%	100%	100%	54.07%	100%	77.80%	66.44%	86.73%
	Age 20–35	40.00%	16.30%	67.70%	85.70%	42.10%	99.60%	85.70%	42.13%	99.64%	40%	16.34%	67.71%
	Age 36–60	55.60%	30.80%	78.50%	100%	90.50%	100%	100%	69.15%	100%	82.22%	67.95%	92%
	Age > 60	0.00%	0.00%	84.20%	91.30%	72.00%	98.90%	0%	0%	84.20%	91.30%	71.96%	98.93%

LC-LN_ Lateral cervical Compartment Lymph nodes; CC-LN: Central Compartment Lymph nodes. Se: Sensitivity; Sp: Specificity; PPV: Positive Predictive Value; NPV: Negative Predictive Value; IC 95%: Confidence Interval 95%.

**Table 6:** Results of multivariate analysis.

MULTIVARIATE ANALYSIS CC-LN				
COEFFICIENTS	Estimate	*p* Value	Lower Bound (O.R.s.)	Upper Bound (O.R.s.)
**(Intercept)**	−15.332	0.995	0	∞
**BMI**	−0.01	0.885	0.868	1.13
**Cytology (Tir 4)**	15.547	0.995	0	∞
**Cytology (Tir 5)**	16.657	0.994	0	∞
**Cytology (Tir 3A)**	14.859	0.995	0	∞
**Cytology (Tir 3B)**	14.614	0.995	0	∞
**Maximum FNAB lymph nodes size (mm) on US**	0.009	0.742	0.957	1.063
**Hypoechogenic**	−0.846	0.183	0.123	1.491
**Hyperechogenic**	−15.353	0.995	0	∞
**Hyperechogenic spot**	−0.363	0.517	0.232	2.086
**Mixed composition**	0.267	0.763	0.23	7.407
**Solid composition**	−1.583	0.051	0.042	1.006
**MULTIVARIATE ANALYSIS LC-LN**				
**COEFFICIENTS**	Estimate	*p* Value	Lower Bound (O.R.s.)	Upper Bound (O.R.s.)
**(Intercept)**	−17.346	0.998	0	∞
**BMI**	−0.032	0.689	0.826	1.134
**Cytology (Tir 4)**	15.912	0.998	0	∞
**Cytology (Tir 5)**	18.14	0.998	0	∞
**Cytology (Tir 3A)**	0.166	1	0	∞
**Cytology (Tir 3B)**	16.466	0.998	0	∞
**Maximum FNAB lymph nodes size (mm) on US**	0.018	0.553	0.959	1.082
**Hypoechogenic**	−0.111	0.878	0.219	3.66
**Hyperechogenic**	−16.346	0.998	0	∞
**Hyperechogenic spot**	−0.745	0.264	0.129	1.754
**Mixed composition**	0.567	0.594	0.219	14.192
**Solid composition**	−1.107	0.225	0.055	1.976
**PERFORMANCE METRICS LC-LN**				
**Accuracy**	0.838			
**AUC**	0.803			
**Sensitivity**	0.333			
**Specificity**	0.954			
**Precision**	0.625			
**PERFORMANCE METRICS CC-LN**				
**Accuracy**	0.775			
**AUC**	0.775			
**Sensitivity**	0.56			
**Specificity**	0.873			
**Precision**	0.667			

BMI: Body Mass Index; Tir 3A/B: risk-indeterminate nodule; Tir 4: suspicious of malignancy nodule; Tir 5: malignant nodule US: ultrasonography; AUC: Area Under ROC Curve; O.R.s.: Odds Ratio scale.

## Data Availability

The data presented in this study are available upon request from the corresponding author. The data are not publicly available due to patients’ data confidentiality.
